# **Acinetobacter baumannii** infection in a medical intensive care unit: The impact of strict infection control

**DOI:** 10.7196/AJTCCM.2019.v25i1.239

**Published:** 2019-04-12

**Authors:** A M Aboshakwa, U Lalla, E M Irusen, C F N Koegelenberg

**Affiliations:** Division of Pulmonology, Department of Medicine, Stellenbosch University and Tygerberg Academic Hospital, Cape Town, South Africa

**Keywords:** Acinetobacter baumannii, nosocomial, infection control

## Abstract

**Background:**

*Acinetobacter baumannii* is a waterborne organism that preferentially colonises aquatic environments. Infections usually involve organ systems that have a high fluid content. Multidrug-resistant (MDR) *A. baumannii* is recognised to be among the most difficult antimicrobial-resistant Gram-negative bacilli to prevent and treat in the nosocomial setting.

**Objectives:**

To determine the utility of concomitant implementation of a strict antimicrobial stewardship programme and comprehensive infection control measures to control MDR *A. baumannii* in a medical intensive care unit (ICU).

**Methods:**

We retrospectively compared the relative incidence of *A. baumannii* infections in our unit over a 1-year period before (2012) and after (2016) the implementation of strict infection control bundles. Patients with *A. baumannii* infections were identified using the microbiology database of the National Health Laboratory Service’s central data warehouse. The total number of admissions and clinical data were derived from the ICU registry.

**Results:**

*A. baumannii* was isolated from 43/263 patients (16.35%) in 2012 compared with 37/348 patients in 2016 (10.63%, p=0.03; relative risk reduction=35%). We found almost 100% sensitivity to colistin and tigecycline, but 90% resistance to carbapenem antibiotics.

**Conclusion:**

The introduction of strict infection control bundles had a statistically significant and clinically meaningful impact on the incidence of nosocomial *A. baumannii* infection in the medical ICU.

## Background

*Acinetobacter baumannii* is a pleomorphic aerobic Gram-negative bacillus that is commonly isolated from the hospital environment and hospitalised patients.^[1]^ It is a waterborne organism that preferentially colonises aquatic environments and, in hospitalised patients, is often cultured from sputum or respiratory secretions, wounds, urine, and irrigating and intravenous solutions.^[2]^

*A. baumannii* has a low virulence but can cause infection in patients with organ transplants and febrile neutropenia. Most *A. baumannii* isolates recovered from hospitalised patients, particularly those recovered from respiratory secretions and urine, represent colonisation rather than infection; however, care must be exercised in making that determination. Multiple factors increase the risk for acquiring an *A. baumannii* infection in the hospital setting, such as prior antibiotic exposure, intensive care unit (ICU) admission, use of a central venous catheter, mechanical ventilation or haemodialysis.^[2,3]^

*A. baumannii* infections usually involve organ systems that have a high fluid content (i.e. the respiratory and urinary tracts, cerebrospinal fluid and peritoneal fluid). These infections may occur as outbreaks rather than isolated cases of nosocomial infection. Infections may also complicate continuous ambulatory peritoneal dialysis or cause catheter-associated bacteriuria.^[4]^

Multidrug-resistant (MDR) *A. baumannii* is recognised to be among the most difficult antimicrobial-resistant Gram-negative bacilli to prevent and treat. Increasing antimicrobial resistance among *A. baumannii* isolates has been documented, although definitions vary in the literature; the most widely used definition of MDR *A. baumannii* is resistance to more than three classes of antibiotics.^[5]^

Antimicrobial resistance greatly limits the therapeutic options for patients who are infected with this organism, especially if isolates are resistant to the carbapenem antibiotics. Therapeutic options for the treatment of MDR *A. baumannii* infection are thus limited. The development or discovery of new therapies, well-controlled clinical trials of existing antimicrobial regimens and combinations, and greater emphasis on the prevention of healthcare-associated transmission of MDR *A. baumannii* infection are essential.^[6]^

Only a few studies have been performed to assess *A. baumannii* prevalence and resistance in the ICU setting. National awareness of infection control and judicious antimicrobial use is required to overcome this burden.^[7]^ The US Institute for Healthcare Improvement has developed the concept of ‘bundle’ implementation in healthcare to facilitate the clinician’s ability to deliver bedside care more reliably and effectively.^[8]^

A ‘bundle’ is a group of evidence-based care components for a given disease, which, when executed together, may result in better outcomes than if implemented individually.^[8]^ Concomitant implementation of strict antimicrobial stewardship programmes and comprehensive infection control measures have contributed to controlling endemic MDR *A. baumannii* effectively in the ICU setting.^[9]^

Bundles, including those for central line-associated bloodstream infection (CLABSI), ventilator-associated pneumonia (VAP) and catheter-associated urinary tract infection (CAUTI), were introduced at Tygerberg Academic Hospital in 2013. In addition, an antibiotic stewardship programme commenced in the same year.

The primary objective of this study was to compare the relative incidence of *A. baumannii* in the adult medical ICU of Tygerberg Academic Hospital before and after the introduction of the various bundles. The secondary aim was to determine the antimicrobial susceptibility of *A. baumannii* during these study periods.

## Methods

### Study description and population

In this retrospective and analytical study, we compared the relative incidence of positive *A. baumannii* culture(s) in two 1-year periods (2012 and 2016) in the medical ICU of Tygerberg Academic Hospital. Patients with *A. baumannii* isolates were identified using the microbiology database of the National Health Laboratory Service’s central data warehouse. In addition, the ICU registry was used to determine the total number of ICU admissions for 2012 and 2016. All patients older than 13 years from whom *A. baumannii* was isolated (from any site) were included. Patients younger than 13 years and those with incomplete patient data were excluded. Ethical approval for this retrospective analysis was granted by the Stellenbosch University Research Ethics Committee. This approval included a waiver of consent owing to the retrospective nature of this study.

Bundles for CLABSI and VAP were introduced in our unit in 2013. Personnel completed a training programme that included bedside training and didactic lectures on the theory and implementation of the bundles (including the introduction of checklists). Bundle compliance was assessed by trained staff four times per day (according to further checklists) and reported monthly. The bundles complemented the existing antibiotic stewardship interventions, which included antibiotic authorisation and weekly ICU rounds by infectious disease specialists.

### Data collection and processing

The source of *A. baumannii* isolates (sputa, blood, tissues, body fluid and catheters) during admission to our unit and antimicrobial susceptibility patterns were captured. Data obtained included patient demographics, comorbidities, length of stay, admission diagnosis and outcome. The relative incidence over the study period was calculated as the number of patients with *A. baumannii* isolates for a specific year relative to the total number of admissions for that year. No patients were routinely screened for *A. baumannii* during the two periods investigated.

### Statistical analysis

Demographic data with a normal distribution, such as age, gender and race, are reported as means with standard deviations (SD). Data that were not normally distributed are reported as medians with interquartile ranges. Fisher’s exact test was used for categorical variables. Statistical significance was regarded as p<0.05 unless otherwise stated. Continuous variables are presented as mean (SD).

## Results

A total of 263 patients (115 male) were admitted in 2012. This group had a mean age of 39.7 (15.0) years and a mean APACHE score of 15.4 (9.3). In 2016, a total of 348 patients (n=142 male) were admitted, with a mean (SD) age of 39.2 (16.1) years and a mean APACHE score of 18.6 (10.2).

*A. baumannii* was isolated from 43/263 patients (16.35%) in 2012, and 37/348 patients in 2016 (10.63%, p=0.03; relative risk reduction=35%). Bundles had to be observed in 14 patients to prevent one infection.

**Fig. 1:**
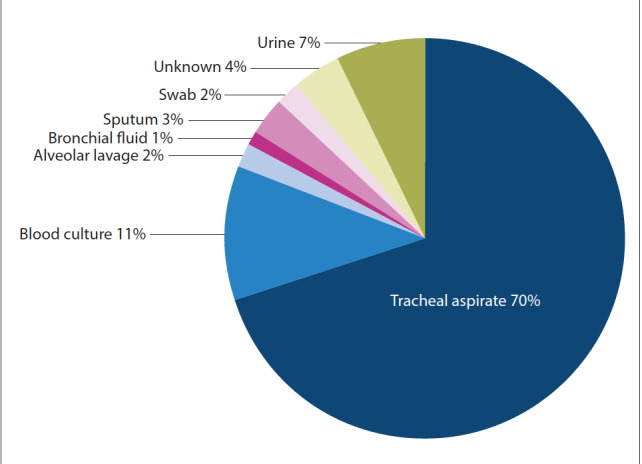
Sources of positive cultures in 2012.

**Fig. 2:**
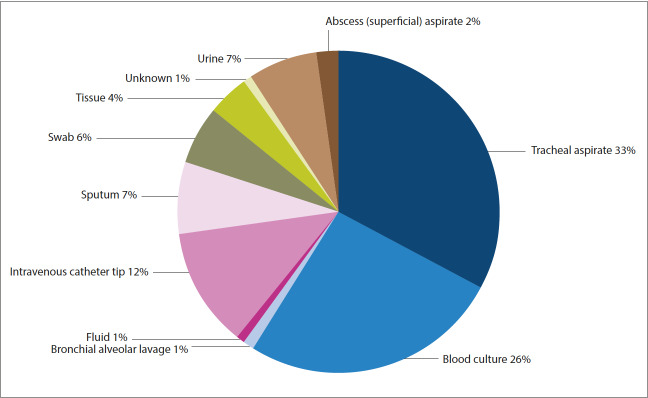
Sources of positive cultures in 2016.

The sources of the positive cultures are summarised in Fig. 1 and Fig. 2 for 2012 and 2016, respectively. The tracheal aspirate was a major source in both years, with a rate of 70% in 2012 and 33% in 2016.

**Fig. 3:**
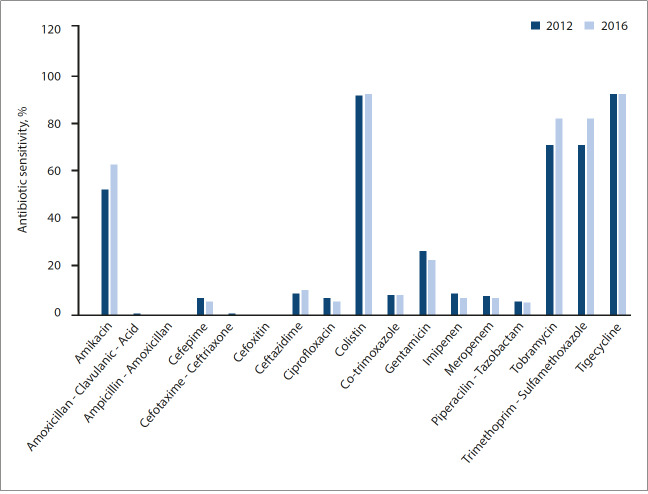
Antibiotic resistance of *Acinetobacter baumannii* cultures in 2012 and 2016.

Antibiotic sensitivities for both 2012 and 2016 are summarised in Fig. 3. In general, *A. baumannii* isolates were almost 100% sensitive to colistin and tigecycline, but only 10% were sensitive to carbapenems.

The mean length of hospital stay was 12.9 (10.9) days in 2012 compared with 10.2 (9.5) days in 2016. In both years, the most common admission diagnosis in patients who went on to develop an *A. baumannii* infection was that of community-acquired pneumonia. The mortality rate of patients with *A. baumannii* infection was 27.9% (12/43) in 2012 and 18.9% (7/37) in 2016.

## Discussion

The introduction of strict infection control bundles had a statistically significant and clinically meaningful impact on the incidence of nosocomial *A. baumannii* infection in the medical ICU. We observed a 5.7% decrease in the isolation of *A. baumannii* following the conscientious introduction of these bundles.

Our observations are comparable to published data. An Italian study showed a significant reduction in the spread of MDR *A. baumannii* among patients during an outbreak.^[10]^ A study that investigated the impact of bundles to reduce sepsis in the ICU showed a significant reduction in the rate of central catheter-related bloodstream infection.^[11]^ Similarly, decreased rates of CAUTI and VAP have been reported in the ICU after the implementation of the respective bundles.^[12,13]^

Overall, the implementation of the various bundles appears to have contributed significantly to reducing the incidence of sepsis in the ICU, especially with regard to resistant bacteria such as *A. baumannii*, and helps to decrease the spread of infection among ICU patients.^[14,15]^

Although the incidence of *A. baumannii* infection is on the rise in developed countries, there is a paucity of data from Africa.^[16–19]^ We found mortality to be as high as 28% in 2012 and 19% in 2016. These figures are comparable to those reported by Trottier *et al*., ^[20]^ who noted a mortality rate of 26.2% in critically ill American patients. Other investigators reported mortality rates of 8% - 43% in developed countries and 33% - 45% in developing countries.^[21–23]^

However, studies generally did not control for confounding risk factors such as age, disease severity and comorbidities. We observed an incidence and mortality rate comparable to that in developed countries, where the control of *A. baumannii* remains a considerable challenge.^[24,25]^ Solutions to the challenge of managing *A. baumannii* are therefore likely to transcend resource and economic boundaries and should include strict attention to infection control and stringent antibiotic stewardship.

Cases of *A. baumannii* resistance to colistin have been reported in a few studies. Such resistance can be acquired through mutations in the *lpxA, lpxC* and *lpxD* genes, leading to complete loss of lipopolysaccharide biosynthesis.^[26]^ Colistin resistance is therefore still considered sporadic. Our study also confirmed an increase in carbapenem resistance. The mechanisms of development of carbapenem resistance in *Acinetobacter* spp. have been attributed to efflux pumps and outer membrane proteins.^[27]^ *A. baumannii* resistance to tigecycline may also develop via the acquisition of efflux pumps.^[28]^

The main strength of our study is that it was performed in a medical ICU with a low staff turnover and a workload that allowed for the strict implementation of the various bundles. Moreover, our policy of replacing central lines (1) in the case of a possible breach in sterility or (2) if a patient was transferred from another hospital may have impacted on the reduction in the rate of CLABSI seen in our unit.

### Study limitations

Most of our ICU patients were referred from secondary hospitals within the drainage area of Tygerberg Academic Hospital. Patients were also intubated, which is known to increase the rate of *A. baumannii* colonisation or infection, independent of the measures implemented. Owing to the wide drainage area, it remains unclear whether patients were colonised or infected with *A. baumannii* prior to their ICU admission at our institution, as no routine screening was performed. Another potential limitation is that we could report the incidence of *A. baumannii* relative only to total ICU admissions during a period and not to the total population at risk. In addition, we could not report on the clinical significance of the positive isolates and whether treatment was instituted or not.

## Conclusion

Despite some limitations, we were able to show that the introduction of strict infection control bundles had a significant and clinically meaningful impact on the incidence of nosocomial *A. baumannii* infections in the medical ICU setting.
